# Asthma among World Trade Center First Responders: A Qualitative Synthesis and Bias Assessment

**DOI:** 10.3390/ijerph15061053

**Published:** 2018-05-23

**Authors:** Hyun Kim, Navneet Kaur Baidwan, David Kriebel, Manuel Cifuentes, Sherry Baron

**Affiliations:** 1 Division of Environmental Health Sciences, University of Minnesota, Minneapolis, MN 55455, USA; navneetbaidwaan@gmail.com; 2 Department of Public Health, University of Massachusetts Lowell, Lowell, MA 01854, USA; david_kriebel@uml.edu; 3 Department of Public Health, Regis College, Weston, MA 02493, USA; manuel.cifuentes@regiscollege.edu; 4 Barry Commoner Center for Health & the Environment, Queens College, New York, NY 11367, USA; sherrybaron@gmail.com

**Keywords:** world trade center, responders, new-onset asthma, bias analysis, review

## Abstract

The World Trade Center (WTC) disaster exposed the responders to several hazards. Three cohorts i.e., the Fire Department of New York (FDNY), the General Responder Cohort (GRC), and the WTC Health Registry (WTCHR) surveyed the exposed responder population. We searched Pubmed and Web of Science for literature on a well-published association between the WTC exposures and asthma, focusing on new-onset self-reported physician-diagnosed asthma. The resulting five articles were qualitatively assessed for potential biases. These papers were independently reviewed by the co-authors, and conclusions were derived after discussions. While, the cohorts had well-defined eligibility criteria, they lacked information about the entire exposed population. We conclude that selection and surveillance biases may have occurred in the GRC and WTCHR cohorts, but were likely to have been minimal in the FDNY cohort. Health care benefits available to responders may have increased the reporting of both exposure and outcome in the former, and decreased outcome reporting in the FDNY cohort. Irrespective of the biases, the studies showed similar findings, confirming the association between WTC exposure and self-reported physician-diagnosed asthma among responders. This suggests that health data gathered under great duress and for purposes other than epidemiology can yield sound conclusions. Potential biases can, however, be minimized by having validated survey instruments and worker registries in place before events occur.

## 1. Introduction

First responders, clean-up workers, and volunteers involved in rescue and recovery following the attack on the World Trade Center (WTC) on 11 September 2001 suffered from extreme hazardous physical, chemical and emotional conditions [1,2]. Shortly after 9/11, local clinicians and researchers began to screen responders, workers and volunteers (hereafter called “responders”) for health effects via separate but coordinated initiatives. These were: the Fire Department of New York (FDNY) that created a program to screen firefighters and emergency medical service (EMS) workers employed by the FDNY [3,4]; and the New York/New Jersey World Trade Center Clinical Center Consortium (hereafter called the General Responder Cohort (GRC)) established to monitor workers and volunteers who performed search, rescue and recovery (excluding FDNY firefighters/EMS) [5]. Detailed cohort profiles have been published elsewhere [2,3,4,5]. A third program began in 2003, with the creation of the New York City (NYC) WTC Health Registry (WTCHR) [6] which surveyed the entire exposed population including both responders and survivors who were living or working in lower Manhattan on 9/11 [7]. The WTCHR conducts periodic health surveys but unlike the other two WTC programs, does not conduct medical screening. All three cohorts collected self-reported information on a list of health conditions that might be related to WTC exposures using questionnaires. Information from clinical examinations prior to 9/11 is also available for the FDNY cohort, but not the other two. Exposure information was gathered by questionnaires, and the specific questions asked varied among the three cohorts.

Multiple research efforts have investigated possible causal links between WTC exposures and a range of health outcomes, e.g., cancer, cardiovascular, and respiratory health outcomes [8,9,10,11,12]. As in any observational epidemiology research, the leap from statistical association to causal inference is challenging. Furthermore, the limitations in the data are even more severe when a disaster is being studied [13,14]. During disasters, exposure data may be limited, as survey protocols are generally implemented without sufficient time for rigorous development, testing, and enumerating the potential target population may be difficult. Consequently, a wide range of potential biases may compromise the validity of epidemiologic investigations conducted in the aftermath of a disaster like the WTC attack [13].

We recognized that the existence of these three WTC cohorts might provide an opportunity to assess the likely impacts of epidemiologic biases, particularly under the challenging circumstances of a disaster. We hypothesized that a comparison of findings from the three cohorts would yield useful information for future disaster epidemiology. We chose to focus on a single exposure-risk association between responder exposures to hazardous material including the dust cloud and new-onset self-reported physician-diagnosed asthma, a well-defined and validated measure for population surveys. Although other measures of asthma were gathered in some studies, the only asthma measure that was common across the cohorts was self-reported physician-diagnosed asthma [6,7,8]. Our goal was to inform future disaster epidemiology studies in which there will likely not be multiple independent sources of health data but there will almost certainly be variable sources of bias.

## 2. Methods

All literature related to WTC exposures and asthma was reviewed in accordance with the Preferred Reporting Items for Systematic Reviews and Meta-Analyses (PRISMA) guidelines [15].

**Figure 1:**
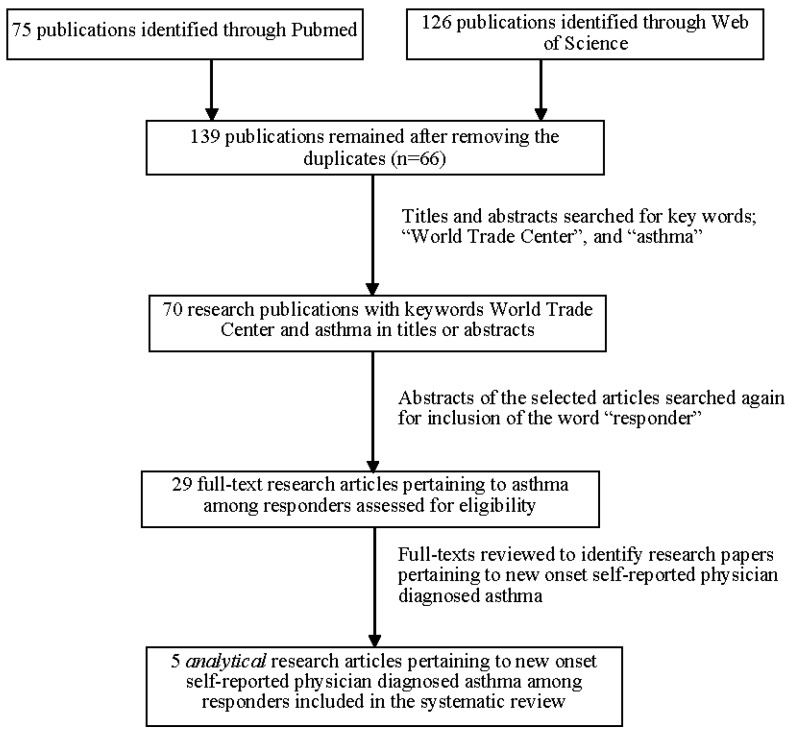
Flow chart for study articles’ inclusion for conducting the qualitative review and bias assessment.

Search Method: We searched “Pubmed” and “Web of Science” databases for English-language publications in the peer-reviewed literature pertaining to asthma among WTC responders. We used the keywords: “World Trade Center” and “Asthma” to retrieve the existing literature on the topic, before specifically focusing on self-reported, physician-diagnosed asthma. This search resulted in 75 Pubmed-based articles, and 126 Web of Science-based articles published between January 2002 and June 2017 (Figure 1).

Exclusion criteria: removing duplicates resulted in 139 publications that were systematically assessed to include those that focused on WTC-associated new-onset, self-reported physician-diagnosed asthma. This was achieved in several steps (Figure 1). An Excel sheet was prepared where basic information on each of the 139 papers was recorded. Further details on the Excel sheet are provided in the Appendix A. An independent reviewer reviewed both the titles, and the abstracts of these publications to identify if both the key words were present in either the title or the abstract. The articles that did not pertain to emergency responders were excluded. Next, articles that focused on WTC disaster associated asthma among the responder population were reviewed (*n* = 29). Full-texts of these 29 stated articles were retrieved, and the aforementioned Excel sheet was used to then summarize necessary details including, authors, title, year of publication, exposures of interest, outcome, variable measurement, main findings, and discussion of the potential biases. Eventually, only five papers [16,17,18,19,20] that reported epidemiologic analyses on self-reported physician diagnosed asthma incidence among responders were included in this qualitative assessment of potential biases. Table 1 provides a description of the study population and exposures and outcomes for each of these articles. In the final step, bias in the reported estimates of the association between WTC exposures and asthma from these five papers were systematically reviewed and evaluated.

Exposure and outcome assessment: the selected studies were evaluated for exposure ascertainment and outcome measurement, along with the measure of association used, and the authors’ discussion/recognition of biases. The primary exposure measure was being a responder, but in addition a variety of different WTC exposure proxies were measured using questionnaires including arrival day and time, duration on the site and being in the dust cloud. These were considered proxies for exposure to dust and gases from the explosion and fires at Ground Zero (Table 1). In all three WTC cohorts, self-reported asthma was measured with the question “has a doctor ever told you that you have asthma?” or a close variant of this.

Qualitative bias evaluation: we used a structured decision process approach to select and define potential biases and then identify their presence. Three key steps were performed: (1) develop a bias analysis worksheet to extract key information from each article; (2) evaluate the extracted information; and (3) confirm potential biases and their presence qualitatively using expert judgment. Three guidelines were obeyed for epidemiologic study review: the Strengthening the Reporting of Observational Studies in epidemiology (STROBE) checklist [21]; the systematic review and evidence integration for literature-based environmental health science assessments [22]; and the PRISMA checklist [23] were reviewed and key bias-related components combined to conduct the bias evaluation. An Excel sheet was used to record information for each of the studies including, authors, year of publication, title, purpose, study cohort/population, study design, exposures of interest, and outcome as listed in Table 2. The potential biases identified a priori and looked for in each paper were: selection biases from self-selection into cohorts; common-method bias from the use of surveys to assess both exposure and outcome; and differential reporting bias because of incentives and disincentives to report either exposure or outcome or both. Each of the authors independently assessed each article and then shared notes and as a group discussed and reached consensus, if there were divergent assessments.

## 3. Results

### 3.1. Study Populations and Key Findings from the Five Studies Included in This Review

Overall, the direction and strength of association between WTC exposure and asthma reported by the five papers were consistent across the articles. The results are briefly summarized below and findings from the four studies that reported effect measures are shown in Table 3.

FDNY: Webber and colleagues (2011) [18] reported a strong association between asthma incidence and the reported time of arrival at Ground Zero among the firefighters (Table 3). Those who were among the first to arrive on the morning of 9/11 were more than three times as likely to develop asthma as those who arrived three or more days later.

GRC: Wisnivesky and colleagues (2011) [19] created four exposure groups based on combinations of time working at the WTC site and working in the dust cloud and/or working on the pile of debris. No summary measure of exposure and outcome association was provided but the survival curves showed an increasing risk of asthma across the exposure groups. Kim et al. (2012) [20] hypothesized that the cohort would have a higher post-9/11 asthma prevalence than expected based on standard national population data (National Health Interview Survey), as suggested by previous researchers [24,25]. The standardized morbidity ratio comparing post-9/11 asthma prevalence among WTC responders compared to national prevalence was twice as high both for men and women. The consistency of this finding with the other studies using internal comparison groups adds to the overall robustness of the result.

WTCHR: there were several different qualitative measures of dust exposure used by Wheeler and colleagues (2007) [16] to evaluate the association with asthma. They also reported a higher risk among the earliest to arrive at the site using different time categories than Webber et al. (2011) [18]. The consistency of results among these different self-reported exposure measures lends strength to these findings, despite the absence of any objective measurements.

**Figure 2:**
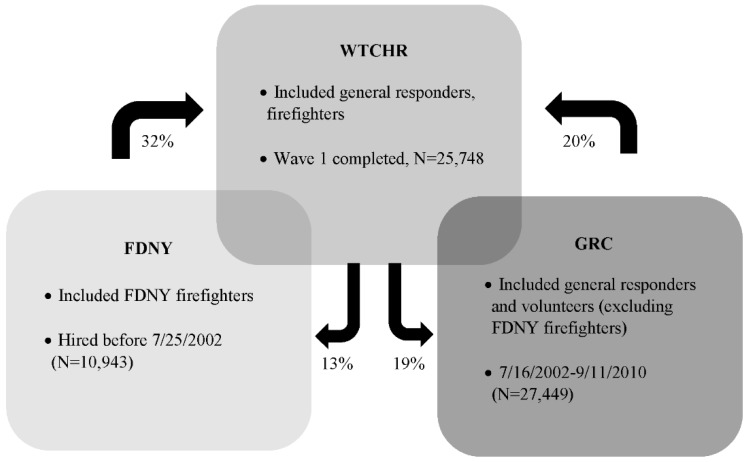
Schematic overview of three WTC cohorts (arrows and percentages indicate the proportion of overlap between cohorts).

Interpretation of the above results should take into consideration the fact that there was modest overlap between cohort memberships, although each study gathered its own data using different methods [26]. Approximately 19% of the WTCHR also participated in the GRC, while approximately 13% of the WTCHR also were included in the FDNY cohort (Figure 2).

### 3.2. Selection Bias

In cohort studies, selection bias occurs when selection into or out of exposure groups is determined by the health outcome, or the potential determinants of health in a cohort/population [25]. There are a large number of mechanisms by which this might have occurred in the WTC studies due to the differences in the design, conduct and goals of the three cohorts. The cohorts probably were affected by different types of selection bias.

Potential for selection bias at enrollment to WTC program: a previous study [19] provided estimates of the total size of the WTC rescue and recovery worker population. The authors suggested that the number of such responders was over 50,000, and there are more than 40,000 WTC responders enrolled in the GRC and FDNY cohort. The WTCHR during its planning stage conducted an extensive assessment of eligible population sizes and composition, resulting in the most authoritative estimate of the different eligibility groups [27,28,29]. If, as these estimates suggest, the surveyed population is close to the true size, these two cohorts combined have been assessing a large proportion of the responders [19]. Next, almost all (*n* = 11,336, 93%) FDNY firefighters [30] who responded to the WTC, participated in the FDNY cohort since it was an employment requirement. On the other hand, enrollment into the GRC [5] and WTCHR was voluntary but associated with extensive outreach efforts. Enrollment into the WTCHR occurred in 2003–2004 and was then closed but during the enrollment period WTCHR staff in collaboration with its various advisory committees developed extensive and systematic sampling and follow-up strategies that would maximize both participation and data quality. Enrollment into the GRC was also extensive and coordinated through labor unions, employers and community-based organizations and remains open up to the present time, allowing participants to enter or exit the cohort at any time [31].

As an open cohort, the GRC may be affected by differential selection into or out of the cohort over time based on both health and exposure status. Also, the benefits associated with enrollment into the GRC have changed over time as the program has expanded to provide additional services [8]. The pattern of first enrollment into the program mirrors this change in benefits showing a spike in enrollment in 2006 and 2007 [19,20]. However, the comparability in the incidence and prevalence of health outcomes to the FDNY cohort which did not have this benefit-related phenomenon suggests that the impact of this selection bias due to delayed enrollment into the GRC is small. Specifically, those with health problems did not enroll disproportionately in the GRC compared to the FDNY cohort [19].

Potential for selection bias at follow-up: selection bias at follow-up becomes most important when there is a latency between exposure and disease incidence and there is differential loss to follow-up over time across exposure and/or outcome groups. While the highest incidence of asthma occurred in the initial post exposure period, studies have demonstrated persistent though modest increases in asthma incidence through 2006 for the WTCHR [17] and through 2010 for the GRC. The GRC had a loss to follow-up at each subsequent visit; 71%, 61%, and 55% respectively after the first, second, and third visit [19]. Despite this, the pattern of WTC exposures and asthma association was similar over time in the studies included as indicated in Table 3, suggesting that the declining participation rate did not introduce important bias. At each additional wave of the WTCHR surveys, participants could also opt out of participation. The WTCHR assessed potential selection bias on follow-up by comparing follow-up rates and asthma incidence rates for participants who “self-identified” compared to participants who were “list-identified,” defined as a responder identified and recruited to the WTCHR because their name appeared on a list of responders [17]. Follow-up rates were lower, and asthma incidence rates were higher for list-identified compared to self-identified cohort members. One might have expected the reverse—that the self-identified would be sicker than the list-identified if responders were enrolling to gain access to medical care [17]. Self-identified may also be more committed to the program and hence may have lower drop-out rates than the latter. A separate analysis of response bias by the WTCHR for participants in wave 2 (2005–2006) and wave 3 (2010–2011) found that the associations between exposure levels and lower respiratory symptoms was not affected by participation status between study waves [32]. The FDNY cohort provides a useful contrast here, because participation was required and nearly universal. The only real opportunity for loss to follow-up was among retirees for whom follow-up was voluntary; but the retirees’ loss to follow-up was small [33].

### 3.3. Differential Surveillance Bias

There was a heightened awareness among physicians in the NYC area about the potential respiratory effects of exposure to the WTC dust. In addition, both the FDNY and the GRC had access to free medical care for WTC-covered conditions. While this was true for both the FDNY as well as the GRC, different responder groups may have experienced different factors that may have acted to either motivate or discourage them from reporting health issues. For example, a diagnosis of asthma might negatively impact firefighters’ medical clearance to perform all duties while the same diagnosis could provide needed treatment at no cost to an unemployed construction laborer. A study [34] assessed the potential bias of self-reported physician diagnosed versus FDNY medical record-confirmed physician diagnosed asthma and found evidence for the association between WTC exposure and asthma incidence using both measures of outcome. The strength of the association was somewhat less using self-reported asthma (odds ratio (OR) = 1.4 compared to 3.3) which may indicate potential reporting bias that may have exaggerated the self-reporting of diagnosed asthma. It is possible that economic factors (benefits, compensations) may have increased self-report of exposure and outcomes in the GRC and WTCHR self-identified participants, while it should have been of minimal impact in the FDNY cohort due for previously discussed reasons.

### 3.4. Exposure and Outcome Misclassification

Exposure misclassification: exposure information in epidemiologic studies can be either qualitative, semi-quantitative or quantitative [35]. Among the selected five studies, there were qualitative (in the dust cloud yes/no) or semi-quantitative (duration of work at Ground Zero) assessments of the exposure (Table 1). There were no studies with high-quality quantitative exposure information with objective measurements. This is not surprising given the extraordinary nature of the disaster. Qualitative and semi-quantitative measures can be quite powerful, particularly when the goal is identification of an association and not quantitative risk assessment.

One of the strongest patterns of evidence was the consistency of findings across several different ways of defining exposure (Table 3). In Wave 2 of the WTCHR survey in 2006–2007, the investigators asked five additional questions regarding the experience of being caught in the dust cloud. Brackbill et al. (2009) defined a category of intense exposure as responding positively to any of these questions, and this was associated with a higher risk of asthma incidence compared to no dust cloud exposure. The lower exposure level, “ever” in the dust cloud had an odds ratio of 1.3 (95% confidence interval (CI) = 1.1 to 1.5), while the intense dust cloud exposure group had an OR = 1.5 (95% CI = 1.4 to 1.7). This gradient provides valuable evidence strengthening the case for causality; one could imagine that if the apparent dust cloud–asthma association were due to selective recall or over-reporting of exposure, then the detailed questions would have weakened the association not strengthened it.

Wisnivesky and colleagues (2011) [19] also used multiple questions or dimensions of exposure by combining responses to several questions on intensity and duration of exposure into a four-level scale. This is another good illustration of the rich information from a series of different questions. When results seem to be consistent across different scales, as in this review, they greatly strengthen plausibility.

Outcome misclassification: Except for Webber et al. (2011) [18], the other four papers used self-reported diagnosis of asthma exclusively. Self-reports of health outcomes among workers are known to have a low/medium level of sensitivity and specificity [36]. Given this expected pattern in self-reporting of asthma outcomes among workers, we could expect more false positives and less false negatives for asthma among WTC responders. In this context, some participation in some cohorts (GRC, WTCHR) provided access to benefits, such as free treatment, which may have increased the sensitivity and, simultaneously decreased the specificity. If this occurred, it would result in lower accuracy and increased misclassification, biasing the associations closer towards the null. FDNY, on the contrary, may have had disincentives to report asthma given that firefighters have to maintain good health conditions to keep their job. These may have resulted in decreased sensitivity and a slight increase in specificity, with a higher chance of misclassification. However, it is reassuring to note that Weakley et al. (2013) [33] confirmed that FDNY physician-diagnosed and self-reported asthma diagnosis were in agreement, suggesting that the observed association, at least in the FDNY study, was closer to the truth.

## 4. Discussion

The WTC disaster, like most major disasters, created many challenges for public health researchers and planners. The potential exposures were massive yet difficult to characterize; the exposed population was large, diverse and challenging to enumerate; and providing optimal exposure monitoring and protection for the large emergency response and recovery workforce was problematic. Epidemiologic investigations following disasters help guide public health activities and, by necessity, disaster-related health studies are different from classic etiologic research. An immediate and essential goal of disaster epidemiology is to determine whether there has been sufficient exposure to known risk factors to cause an elevated incidence of adverse health outcomes. Following the WTC disaster, there is evidence that those responders exposed to a wide range of dusts and combustion by-products are at elevated risk of asthma. The important question that we believe the studies reviewed here have answered is: “was the exposure to the WTC dust cloud sufficiently intense that it increased the risk of asthma in responders?” Put another way, these studies need not accurately quantify the magnitude of the risk of asthma among responders in order to be useful. For this reason, we interpret the qualitative consistency of the findings (Table 3) to be strong evidence that the elevated risk of asthma among responders is real, and less likely the result of biases. We would be reluctant to use these studies to estimate exactly how large an increase in asthma risk occurred, however.

Bias is one of the most significant challenges in epidemiology [37]. Avoiding and controlling potential biases are central to the design and conduct of epidemiologic studies, including disaster epidemiology. For WTC, different cohorts were set up to document the exposures and health consequences of participants [38]. These cohorts have different strengths and weaknesses and this provided an opportunity to learn from comparisons among the results [39]. Although there was some overlap in cohort membership as noted above, the data in each study were gathered using separate methods and at separate points in time—they were gathered at medical monitoring encounters in the case of the FDNY and GRC program participants, while the WTCHR used telephone or mailed surveys. Thus, while it would not be appropriate to conduct a simple meta-analysis using these as three independent estimates of an effect, our purpose was instead to use the three studies as an opportunity to compare various sources, and strengths of biases. For this purpose, the differences in data collection methods were more important than the modest overlap in participants and we believe that the comparisons of the three studies’ results described here were not importantly influenced by the modest membership overlaps.

Following a disaster, it is important that those exposed be rapidly identified and enrolled in a registry to minimize potential biases resulting from selective enrollment of the sickest, the healthiest or the most exposed, among the exposed cohort. While the WTC cohorts had well-defined eligibility criteria, they lacked information about the entire exposed population. There could also have been non-participation among those identified and there still exists some confusion if those who participated were the sickest or the healthiest. However, such non-participation will generally only affect risk estimates if it depends on both the exposure and the outcome. Researchers have suggested identifying all those who may have been exposed to the disaster [40]. However, in such complex disasters, like the WTC this may not be feasible.

While there are many other measures which WTC researchers have used to capture asthma cases, such as use of symptoms [41,42,43,44,45], we limited our analysis just to the one measure of self-reported physician diagnosis. We purposely selected this clearly defined and commonly used survey question to exemplify our approach to bias assessment. A similar approach could be applied to examine bias using other measures to capture potential asthma cases, but this is beyond the scope of this manuscript. For example, there is the potential for misclassification of asthma when relying on reports of respiratory symptoms that share similar symptom complexes with other related respiratory conditions such as reactive airways dysfunction syndrome or chronic obstructive pulmonary disease. In addition, if the quality of clinical information is different by cohorts, it can be a source of differential misclassification between cohorts.

In the analysis phase, analytical techniques can be used to minimize the resulting bias. One such method is the Heckman model, a sample selection bias analysis in cases of non-random population selection when the exposure and outcome relation differs between the selected and non-selected populations [46]. Selection and information biases are created during data collection and it is better to minimize these earlier rather than trying to make corrections in the analysis. However, because disasters are unpredictable, one potential solution would be to obtain detailed information about potential indicators of bias and later adjust for those bias effects. 

The numbers of studies in the review were limited and some of the coauthors of this analysis were involved in some of the studies we reviewed, which may have influenced the qualitative evaluation of biases. However, all relevant studies were included and all biases considered by the study authors were included in this report. Study authors also included research personnel with no previous connection to the WTC research program and the authors reached consensus on all findings.

## 5. Conclusions

It is likely that there were biases in the WTC studies of asthma incidence when defined as self-reported physician diagnosed, but on balance the observed associations across all five studies were similar in direction and strength, thus suggesting that biases were either modest in effect or worked in different directions with little net effect. While it is possible that biases similar in intensity and direction could explain this, these findings need to be combined with a larger discussion regarding causality (biological plausibility, temporality, and consistency). There are clear sources for non-differential misclassification of exposure and outcome, which would likely have reduced the association’s magnitude, but also not-so-clear non-differential misclassification, possibly acting in the opposite direction. On balance, the most likely explanation for the findings is that there was an effect of WTC exposures on asthma incidence.

Future disaster epidemiology can be improved and potential biases minimized by having validated survey instruments and worker registries in place before events occur. Biases will never be completely eliminated, however, and this unique opportunity to compare three different perspectives on the same disaster provides reassurance that health data gathered under great duress and for purposes other than epidemiology can yield fundamentally sound conclusions.

## Figures and Tables

**Table 1:** Summary of the three study cohorts.

World Trade Center (WTC) Cohort					
	General Responders		Fire Department of New York (FDNY)	WTC Health Registry	
**Reference**	Wisnivesky et al., 2011 [19]	Kim et al., 2012 [20]	Webber et al., 2011 [18]	Wheeler et al., 2007 [16]	Brackbill et al., 2009 [17]
**Study population**	Search, rescue, recovery, cleanup workers and volunteers except FDNY employees		Firefighters and emergency medical service (EMS) workers of FDNY	Search, rescue, recovery, cleanup workers and volunteers, including FDNY employees	
**Participants, no.**	27,449	20,834	10,943	25,748	19,788
**Inclusion period**	Participated between 07/16/2002 and 09/11/2010	Participated between 07/16/2002 and 12/31/2007	Hired before 07/25/2002	Completed Wave 1 (2003–2004)	Completed both Wave 1 and Wave 2 (2006–2007)
**Cohort type**	Open cohort since 7/16/2002		Closed cohort limit to exposed to WTC	Closed cohort	
**Follow-up frequency**	Every 12–18 months		Every 12–18 months	Four Waves with unfixed period between Waves	
**Study design for the analysis**	Longitudinal	Repeated cross-sectional	Longitudinal	Retrospective cohort	Retrospective cohort
**Asthma ascertainment**	Self-reported physician diagnosed asthma				
**Verification with medical chart**	No	No	Yes	No	No
**Comparison group**	Internal comparison	External comparison with NHIS	Internal comparison	Internal comparison	Internal comparison
**Type of measure**	Cumulative incidence	Prevalence/Incidence			
**Measure of association**	Cumulative incidence ratio	Standardized Morbidity Ratio	Odds Ratio		
**Exposure variables**					
**Arrival on the site**			Day/Time	Day	Day/location
**Duration**			Months at the site	Days at the site	Days at the site
**In the dust cloud on 9/11**				Yes/No	Intense/some/no
**Work on the pile**			Yes/No	Ever/never	Yes/No on 9/11
**Use respiratory protection**				Initial date and amount delay of masks and respirators use	
**Other exposure variable**	Days in the cloud and/or on the pile	WTC responder versus general population			

Note: blank cells for exposure variables mean that measure of association was not presented in the study.

**Table 2:** Checklist for qualitative bias evaluation.

Key Component for Bias Evaluation
Study design
Exposure measurement
Outcome measurement
Measured association
Lag period
Control selection bias (no-controls and no-exposed)
Self-selection bias/response bias
Loss to follow-up
Differential surveillance/diagnosis/referral
Recall bias/reporting bias
Interviewer bias
Healthy worker effects
Overmatching
Ecologic exposure misclassification
Differential (systematic)/ non-differential (random) misclassification
Magnitude and direction
Probable unmeasured confounders

**Table 3:** Summary of quantitative evidence for the association of WTC exposures and asthma.

Reference	Exposure Variable	Asthma Variable *	Population **	Contrast	Effect Estimate ***	95% CI †
Kim et al., 2012 [20]	Being a WTC responder	Self-reported	GRC—male	Responder vs. U.S. population	SMR = 2.4	2.2–2.5
		Self-reported	GRC—female		SMR = 2.2	2.0–2.5
Webber et al., 2011 [18]	Time of arrival at WTC site	Self-reported	FDNY	a.m. 9/11 vs. 9/14 or later	OR = 3.3	2.4–4.8
		Clinically confirmed	FDNY		OR = 1.4	1.0–2.1
Wheeler et al., 2007 [16]		Self-reported	WTCHR	9/11 vs. 1/1/2002 or later	OR = 1.8 ††	1.2–2.7
	Duration on site	Self-reported	WTCHR	> 90 days vs. 1–7 days	OR = 1.7 ††	1.4–2.1
	Worked in dust cloud	Self-reported	WTCHR	ever/never	OR = 1.3 ††	1.1–1.5
Brackbill et al., 2009 [17]		Self-reported	WTCHR	“intense” dust cloud vs. no	OR = 1.5 ††	1.4–1.7
Wheeler et al., 2007 [16]	Worked on pile	Self-reported	WTCHR	ever/never	OR = 1.3 ††	1.1–1.5

* 12-month incidence, asthma incidence (GRC, FDNY) or post-9/11 new onset (WTCHR); ** GRC = General Responder Cohort; FDNY = Fire Department of New York; WTCHR = World Trade Center Health Registry; *** SMR = Standardized Morbidity Ratio; OR = Odds Ratio; † 95% confidence interval; †† adjusted for the other exposure variables; Note: missing from this table: Wisnivesky et al., 2011—there is no quantitative effect measure.

## Appendix A

Resources: in recent years, several advances have been made in the field of disaster preparedness to try to get out in front of this challenge. For example, after Hurricane Sandy, the National Institute of Environmental Health Sciences implemented a rolling research fund to support research efforts following unpredictable circumstances like natural or made-made disasters (https://grants.nih.gov/grants/guide/pa-files/PAR-13-136.html). Recently a special public health preparedness supplement was published that presented information on several forms of preparedness including that for calamities like earthquakes, influenza pandemic, bioterrorism attacks, and strengthening surveillance systems etc. was also published (https://ajph.aphapublications.org/doi/full/10.2105/AJPH.2017.304039). The Centers for Disease Control and Prevention have also developed disaster-specific assessment tools (https://www.cdc.gov/nceh/hsb/disaster/docs/DisasterDeathSceneToolkit508.pdf). Community Assessment for Public Health Emergency Response provides another toolkit that assists public health professionals and emergency management officials to rapidly determine the health and other basic needs of those affected (https://www.cdc.gov/disasters/surveillance/pdf/CASPER_Toolkit_Version_2_0_508_Compliant.pdf). Also available are resources for mortality surveillance (https://www.cdc.gov/disasters/surveillance/pdf/disaster-mortality-form.pdf), morbidity surveillance (https://www.cdc.gov/disasters/surveillance/pdf/NaturalDisasterMorbiditySurveillanceIndividualForm.pdf), and other disaster specific tools (https://www.cdc.gov/disasters/surveillance/). Additionally, the Centers for Disease Control and Prevention also funds 14 Preparedness and Emergency Response Learning Centers across the United States that train state, local, and tribal public health authorities in the area of public health preparedness and response (https://www.cdc.gov/phpr/perlc.htm).
